# BioMold: A Standardized Template to Optimize Safety and Precision in Poly-L-Lactic Acid Injection Procedures

**DOI:** 10.7759/cureus.103458

**Published:** 2026-02-12

**Authors:** Andrea D Tedesco, Antony P Barbosa

**Affiliations:** 1 Department of Dental Clinic, Federal University of Rio de Janeiro, Faculty of Dentistry, Rio de Janeiro, BRA; 2 Department of Pharmacy, Pontifícia Universidade Católica de Minas Gerais (PUC-Minas), Belo Horizonte, BRA

**Keywords:** biostimulatory injectables, facial regeneration, injection planning, poly-l-lactic acid, standardization

## Abstract

Poly-L-lactic acid (PLLA) is widely used in aesthetic medicine for its ability to induce neocollagenesis and restore facial volume. Although considered a predictable procedure, technical variability during injection, particularly inconsistent volumetric planning and heterogeneous product distribution, may result in nodules or surface irregularities. This technical report introduces and describes a reusable silicone template designed to assist in volumetric planning for PLLA-SCA (Sculptra; Uppsala, Sweden: Galderma) injections. The BioMold (São Paulo, Brazil: TAB Instrumentos Cirúrgicos Ltd) was developed to support clinicians in performing more structured treatment mapping compared with conventional freehand marking approaches. Rather than relying exclusively on linear retroinjection patterns, the device proposes a spatially organized planning strategy based on predefined geometric sectors. By incorporating commonly adopted reconstitution parameters (10 mL total volume: 8 mL sterile water + 2 mL lidocaine) and the frequently used dose reference of approximately 0.2 mL/cm², the template is intended to help estimate treatment area and injection volume per vector.

The device is available in three base sizes (3 cm, 4 cm, and 5 cm), corresponding to volumetric areas of approximately 7.2-12 cm² and suggested injection volumes of 1.4-2.4 mL. Its trapezoidal geometry and integrated slots allow clinicians to transfer retroinjection pathways directly to the skin, which may support more uniform product distribution compared with conceptual mapping techniques. BioMold represents a standardized physical mapping alternative to subjective freehand planning in PLLA procedures. While clinical performance and outcome impact require future investigation, the device is intended to improve planning reproducibility and procedural organization in biostimulatory treatments.

## Introduction

Injectable biostimulators are popular for treating volume loss and skin laxity by stimulating collagen production and providing long-lasting results. Sculptra (poly-L-lactic acid {PLLA-SCA}; Uppsala, Sweden: Galderma) is a biodegradable polymer composed of poly-L-lactic acid that acts as a deep tissue regenerator, gradually enhancing skin thickness and appearance, with notable clinical improvements lasting up to two years after treatment [1,2]. It was approved in Europe as a dermal filler in 1999, and the United States followed in 2004 for HIV-related lipoatrophy. The FDA later expanded indications to nasolabial folds in 2009 and to fine lines and cheek wrinkles in 2023 [3]. Numerous studies have since confirmed its safety, efficacy, and durability [1,3,4]. Current consensus and recent clinical trials recommend reconstituting the product with 8 mL of sterile water for injection (SWFI), followed by the addition of 1 mL of 2% lidocaine [1,3,4]. Research indicates that reconstituting PLLA-SCA immediately before treatment does not affect its properties or safety profile [1,3,5].

The recommendations for the tolerability of PLLA-SCA suggest using one to two vials per treatment session [4]. A reassessment of the need for further treatment is recommended four to six weeks after the previous session, in accordance with the product’s instructions for use. Overcorrection must be avoided, as the effects develop over time. Since determining the ideal product distribution based solely on the number of vials can be challenging, it has been suggested that an ideal volume of 0.1-0.2 mL/cm² of the reconstituted PLLA-SCA should be used in the treated area [4]. While these guidelines are helpful for injectors, they represent a two-dimensional measurement applied to the three-dimensional structure of facial skin. To enhance patient care, we developed BioMold (São Paulo, Brazil: TAB Instrumentos Cirúrgicos Ltd), an innovative device that assists injectors in planning the optimal and safe volume of PLLA-SCA for treatment areas. This approach is designed to provide personalized care while reducing the risks of adverse events.

## Technical report

Despite clear dosing guidelines for PLLA-SCA injections, no practical bedside tool currently exists to translate mL/cm² recommendations into reproducible injection planning. The BioMold device was therefore developed as an anatomically adaptable and geometrically standardized template intended to reduce injector-dependent variability during PLLA-SCA procedures. Although PLLA-SCA demonstrates consistent regenerative outcomes in clinical studies, delayed adverse events - particularly palpable nodules and surface irregularities - are frequently associated with uneven microparticle distribution caused by inconsistent vector spacing, injection depth, and volumetric estimation [1,3-5]. BioMold addresses this limitation by converting the recommended dose reference (0.2 mL/cm²) into a measurable geometric mapping system that supports more uniform treatment planning.

**Figure 1:**
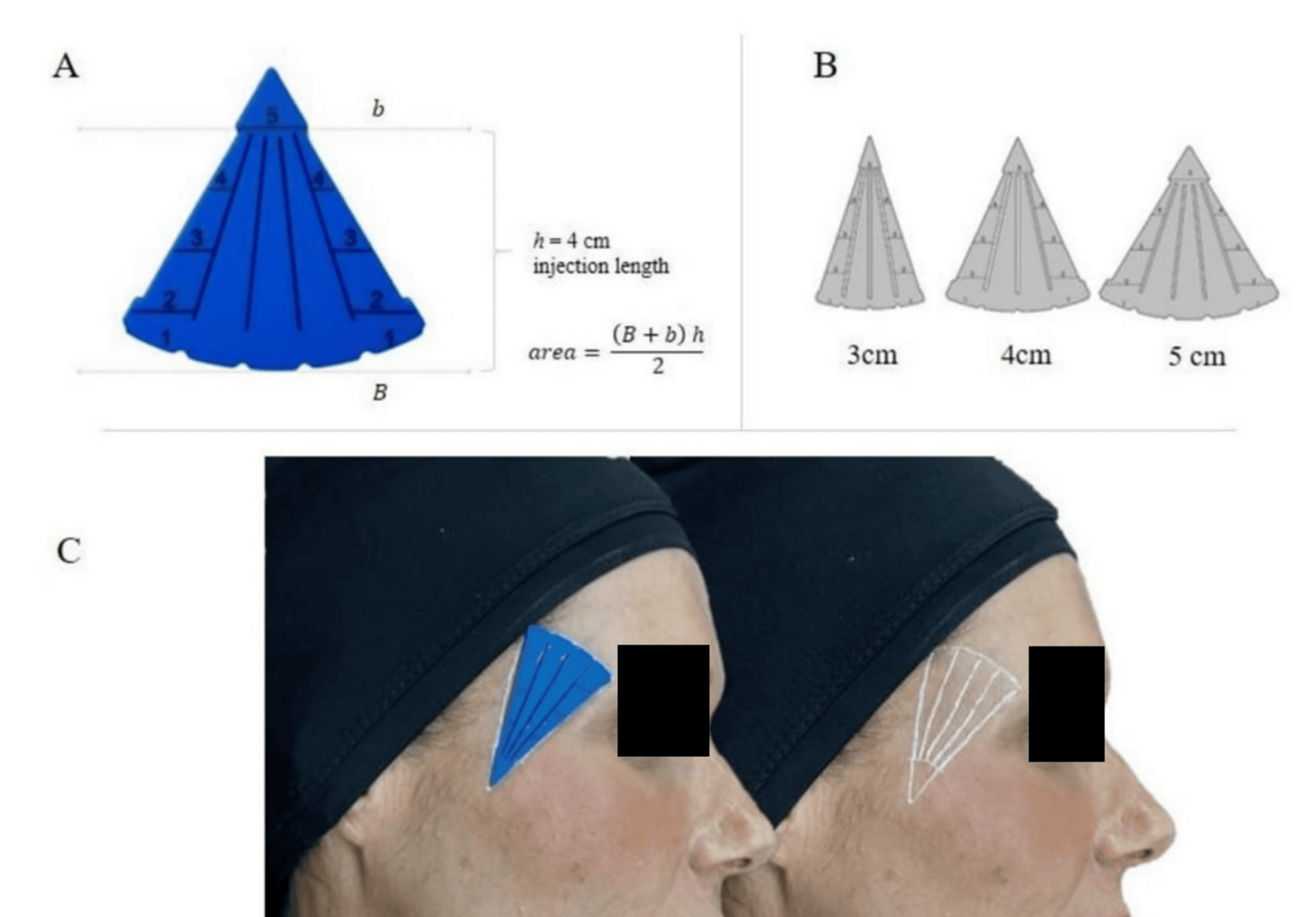
BioMold device, available sizes, and example of vector marking during use. (A) The BioMold device and formula used to calculate the injection area. (B) Three sizes for customizable treatment, with slots for retroinjection vectors. (C) Example of the device in use, showcasing the 4 cm version, before and after drawing the vectors. BioMold (São Paulo, Brazil: TAB Instrumentos Cirúrgicos Ltd)

The conceptual structure of the device is based on transforming the traditional triangular planning area into an effective trapezoidal injection zone. This modification follows established recommendations advising against product delivery within the first centimeter adjacent to the entry point, where superficial placement may increase nodularity risk [4,5]. By excluding this segment, the functional treatment area assumes a trapezoidal geometry, allowing precise surface area calculation using the formula (B+b)×h/2, where B represents the inferior base, b the reduced internal base after exclusion of the non-injection segment, and h the effective retroinjection height. This structure is illustrated in Figure 1, panel A, depicting the BioMold device and its geometric model.

To accommodate different facial anatomies and indications, BioMold is produced in three base sizes (3 cm, 4 cm, and 5 cm), each corresponding to a defined trapezoidal treatment area and proportional injection volume derived from the dosing reference. Table 1 summarizes the effective areas, total treatment volumes, and distribution per injection vector. The smallest model (3 cm base) corresponds to approximately 7.2 cm² and 1.44 mL, whereas the largest (5 cm base) represents 12 cm² and 2.4 mL. Figure 1, panel B, displays the three device versions and slot configurations.

**Table 1:** Calculation details of the BioMold device. *Considering the recommendation of 0.2 mL/cm² in 10 mL reconstitution volume [2]. Here, "B" represents the inferior base and "b" the reduced internal base after exclusion of the non-injection segment.

BioMold (B) size	BioMold (b) size	Trapezoid area	Total volume^*^	Volume per vector^*^
3 cm	0.6 cm	7.2 cm²	1.44 mL	5 vectors of 0.3 mL each
4 cm	0.9 cm	9.8 cm²	1.96 mL	5 vectors of 0.4 mL each
5 cm	1.0 cm	12 cm²	2.4 mL	6 vectors of 0.4 mL each

A key functional feature is the slot-based vector system, which guides the placement of evenly spaced retroinjection markings directly on the skin. Depending on device size, five or six standardized vectors are generated to promote homogeneous distribution. After marking, the device is removed for antisepsis prior to injection (Figure 1, panel C). Standardized spacing reduces localized microparticle accumulation associated with heterogeneous injection patterns and subsequent foreign-body response related to nodule formation [5,6].

BioMold is manufactured from medical-grade silicone chosen for biocompatibility, flexibility, durability, and ease of disinfection. The material conforms to curved facial surfaces while maintaining stability during marking, and its non-porous structure permits cleaning and reuse in accordance with clinical protocols. The device does not replace anatomical assessment or individualized treatment planning; rather, it provides a reproducible geometric reference intended to support procedural consistency in PLLA-SCA applications.

## Discussion

The BioMold device was developed to address a recurring challenge in PLLA-SCA treatments - variability associated with manual injection techniques. Although PLLA-SCA is recognized for its regenerative properties, clinical outcomes remain strongly dependent on uniform particle dispersion within the tissue. Irregular distribution has been associated with localized inflammatory responses and delayed nodules, highlighting the importance of minimizing technique-related variability during injection [1,4,6-8]. In this context, the device provides a structured approach to translate dosing-per-area recommendations into a reproducible clinical workflow.

Histological and clinical studies support the relevance of distribution patterns in the behavior of PLLA. Lemperle et al. and Fitzgerald et al. demonstrated that diffuse dispersion favors progressive collagen formation, whereas clustered deposits increase granulomatous activity and surface irregularities [6,9]. Similar findings by Narins et al. and Fitzgerald and Vleggaar indicate that most late complications are technique-related rather than material-related [10,11]. These observations help explain the difficulty in achieving consistent outcomes using visual estimation alone, particularly across different facial anatomies.

The BioMold should be understood as an adjunctive reference rather than a substitute for clinical judgment. Anatomical assessment, depth selection, and individualized volume adjustments remain the responsibility of the injector. The device provides spatial orientation to support planning but does not determine treatment decisions. Structured planning tools have been proposed in other areas of aesthetic medicine to improve procedural consistency [12,13]. The BioMold follows a similar rationale by offering a quantitative reference aligned with dose-per-area recommendations, aiming to reduce reliance on purely subjective mapping.

From a practical standpoint, the device may be particularly useful in large and relatively flat facial regions (such as the lateral cheeks and preauricular areas), during injector training, and in settings where procedural standardization is desirable across different operators or treatment sessions.

Important limitations must be acknowledged. This report does not include clinical outcome data, complication rates, or patient-reported measures; therefore, no conclusions regarding clinical efficacy or safety improvements can be drawn. Additionally, the device was designed specifically for facial PLLA-SCA use and may require adaptation for other anatomical areas or products. Future comparative clinical studies will be necessary to determine its real-world impact and refine its indications.

## Conclusions

The BioMold device provides a practical and reproducible tool to support volumetric planning and safer procedural planning in PLLA-SCA injection procedures. By standardizing injection vectors and guiding product distribution, the device aims to facilitate more structured treatments while potentially reducing technique-related variability. Although further clinical studies are required to evaluate its performance in real-world practice, BioMold represents an approach toward greater procedural consistency in biostimulatory facial treatments.
